# A new method for ferroresonance suppression in an IEEE 33-bus distribution system integrated with multi distributed generation

**DOI:** 10.1038/s41598-023-30268-w

**Published:** 2023-02-28

**Authors:** Alaa M. Abdel-hamed, Mohamed M. El-Shafhy, Ebrahim A. Badran

**Affiliations:** 1 Electrical Power and Machines Department, High Institute of Engineering, El-Shorouk Academy, Cairo, Egypt; 2 Electrical Engineering Department, Faculty of Engineering, Mansoura University, Mansoura, Egypt

**Keywords:** Engineering, Electrical and electronic engineering

## Abstract

Although integrating a distributed generation (DG) into a distribution system (DS) has several benefits, it may be accompanied by some issues, such as ferroresonance. Therefore, ferroresonance investigations in an integrated DS with multi-DGs have been identified as a research gap. To this end, this paper presents a new method to mitigate ferroresonance in distribution networks, after which ferroresonance in an IEEE-33 bus radial DS integrated with multi-DGs was investigated. Here the RLC shunt limiter is introduced as a method for mitigating ferroresonance, including a design approach for adjusting its dimensions to fit the system. Investigations revealed that this shunt relied on the negative sequence detector to connect it to the system during ferroresonance. Finally, the effectiveness and superiority of the proposed method have been demonstrated by comparing its result with those obtained using other existing ferroresonance mitigation methods used in the literature.

## Introduction

With growing global fears about the depletion of fossil fuels, including the environmental consequences of their use, the adoption of Distributed Generation (DG) has been portrayed as the ideal solution^1^. Notably, the spread of DG has contributed numerous avails to the electrical system, the environment, and consumers^2,3^. Besides, the cost of losses in the electrical network raises consumers’ bills. As a result, DGs are considered a significant benefit to consumers in terms of lowering their costs by reducing electrical system losses^4,5^. Furthermore, renewable DGs contribute to the mitigation of the problem of global warming and Green-House Gases, in addition to reducing emissions^6^^,^^7^. Therefore, it is expected that by 2050, the rated energy generated from renewable energy sources will account for half of the world’s energy electricity^8^. Additionally, DGs supported the expansion of the power market and investments in the electrical grids^9^, they are an excellent solution to transportation line congestion^10^. They are also employed to reduce system power losses, improve power quality, and enhance a system's reliability^11,12^. However, the benefits differ depending on the type of DG. Also, although DGs have numerous advantages, some issues have arisen due to their use. As a result, considerable effort is expended to investigate and resolve most problems. Presently, four types of DGs have been identified. The first type injects active power only, the second type injects both active and reactive power, the third type injects reactive power only, and the fourth type injects real power unless it consumes reactive power^13^. Hence, due to the advantages of this system, several studies have been conducted on the effect of DGs on a network to demonstrate their efforts.

Among these studies, Ref.^1^ described a controller method for improving the synchronization stability of inverter-based DGs into a grid during faulty conditions. Their model was based on the determination of the maximum fixed frequency deflection. However, Ref.^14^ introduced the role of integrating wind-type DGs into the transmission section to reduce the cost of electricity generation and CO_2_ emissions. They also demonstrated the cost of investing in wind energy, including its role in improving the electricity market. In contrast, while Ref.^15^ discussed the methods for integrating DGs with electric vehicles into a Distribution System (DS) to improve system performance metrics, Ref.^16^ devised a methodological approach to determine the optimal power balance between centralized stations and DGs. Moreover, Ref.^17^ presented an algorithm for intensifying a protection system based on distance protection relays at fault conditions in ring grids with a high penetration of DGs. In another study, while Ref.^7^ explained the use of a matrix converter stabilizer to control bidirectional power flow caused by DGs, Ref.^18^ employed DGs to improve the voltage profile of DS. Ref.^19^ also presented a C-type filter design for mitigating the harmonics caused by renewable DGs, whereas, Refs.^19^^,^^20^ presented the contribution of inverter-based DGs to support the dynamic response of a system and frequency recovery responses in the possible shortest time. Then, Ref.^8^ presented the implementation of regenerative DGs in DS, including the procedures for controlling them under low voltage conditions; Ref.^22^ presented improvements in the voltage and frequency of the DS integrated with inverter-based DGs by adjusting the system lines impedance, and Ref.^23^ discussed the use of battery energy storage systems, in conjunction with inverter-based DGs, to improve transient system stability.

From the literature review presented above, the importance of DGs and the great efforts made to maximize their benefit and face their anticipated problems is clear. However, the ferroresonance problem remains a research gap that has not been thoroughly investigated and is almost entirely ignored with DGs, making it a gap point despite the seriousness of this phenomenon. Besides, while previous studies conducted to investigate ferroresonance only focused on this phenomenon from the perspective of the protective element^24^, those on DS with DGs that were interested in ferroresonance only investigated this phenomenon but did not provide mitigation methods^25^^,^^26^. Furthermore, the study, which focused on suppressing the ferroresonance caused by the DGs in the DS, only provided the equivalent circuit of the system, additionally relying on ferroresonance mitigation only after the fault was removed^27^.

Therefore, this paper presents a research gap point: the investigation of ferroresonance in a DS integrated with multi-DGs. To this end, a new method for mitigating ferroresonance in the event of a series fault is proposed. Then, different conditions were presented to investigate ferroresonance in an IEEE 33-bus system integrated with wind DG and capacitors. Finally, the RLC Shunt Limiter (RLC-SL) was proposed as a ferroresonance mitigation element, after which it was compared with other previously used mitigation methods from the literature. The RLC-SL connection is adopted, which relied on a negative sequence detector.

The main contributions of the paper are given as follows:

(i) To investigate the ferroresonance of the IEEE 33 bus distribution system integrated with multi DGs.
(ii) To introduce the (RLC-SL) as a novel technique for reducing ferroresonance and its adjusting procedures.
(iii) The parameter of the proposed scheme is designed and its dimensions, to fit the system, are adjusted.
(iv) To successfully implement the proposed control steps to fast trip the RLC-SL to the grid at ferroresonance, additionally, it separates fast from the system recovery.
(v) To prove the effectiveness of the proposed method by comparison to other existing ferroresonance mitigation methods.

The rest of the paper is organized as follows. “System modeling” section presents the modelling of the IEEE 33 bus system, as well as its integration with capacitors and wind DG on the load side. “Investigation of ferroresonance” section investigates ferroresonance in a variety of series fault conditions on the load side and at the wind DG. “Mitigation of ferroresonance” section introduces the proposed RLS-SL as a new ferroresonance mitigation technique and compares it to some existing ferroresonance mitigation methods. The conclusion of the paper is given in “Conclusion” section.

### System modeling

**Figure 1:**
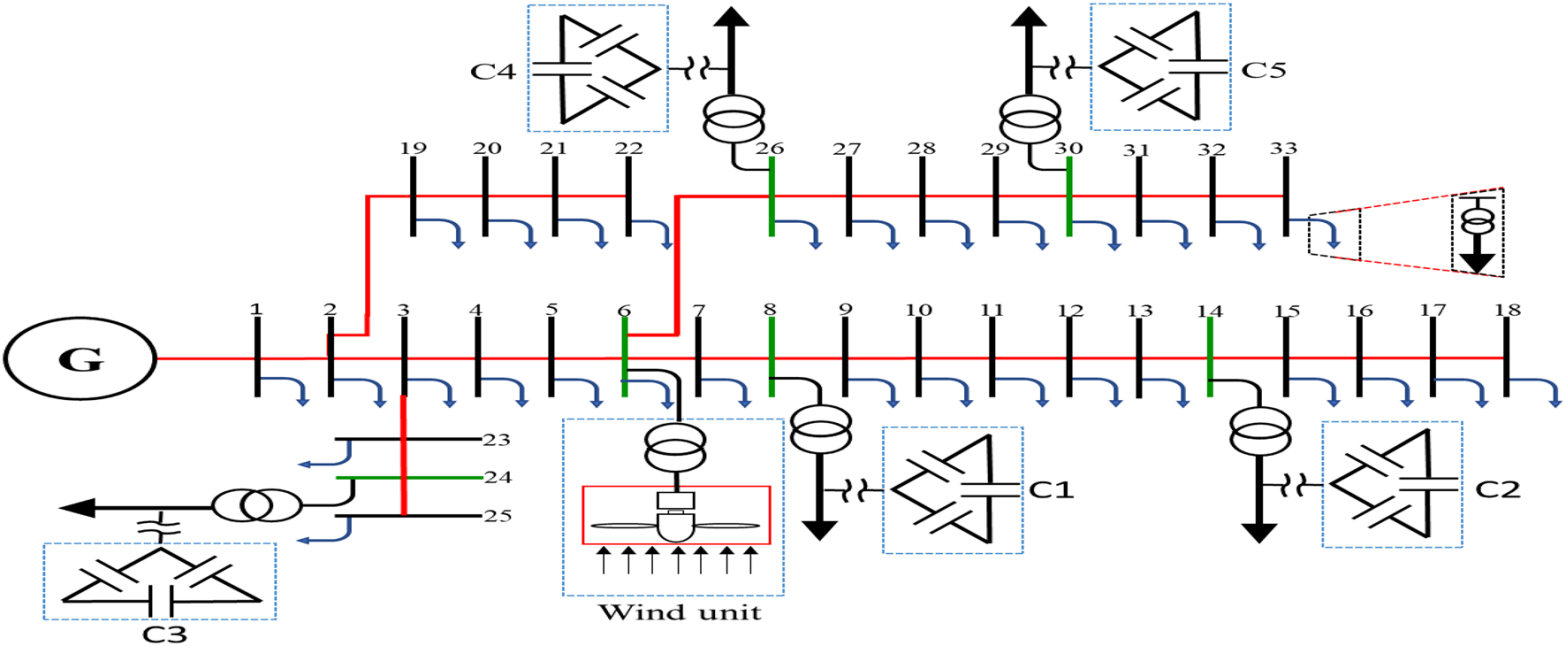
A modified IEEE-33 bus system integrated with DGs.

This section presents a case study on a modified IEEE-33 bus DS penetrated with multiple DGS. After it was built using 33 buses and 32 lines^28,29^, it was simulated using the PSCAD/EMTDC software, with the voltage level of the system being 12.66 kV. Next, it was modified by adding five capacitors (third DG type) and a wind turbine (first DG type) to improve voltage and reduce losses. Figure 1 presents the configuration of the IEEE 33-bus system integrated with DGs. Notably, the capacitors were positioned as close to the loads as possible. As a result, all capacitors were connected at a load voltage of 0.4 kV after a 12.66/0.4 kV distribution transformer, and the wind DG was linked to the system via a 0.69/12.66 kV transformer. Then, the capacitor sizes were determined using Eq. (1)^30^. The sizes and locations of all capacitors and the wind turbine are shown in Table 1^28,31^.

**Table 1:** Data of distribution generations.

Capacitor data				Wind unit data	
Cap. No	Size		Location (Bus)	Size P(MW)	Location (Bus)
	Q (MVAR)	C (mF)			
C1	0.18	2.984	8	1.65	6
C2	0.24	3.9788	14		
C3	0.48	7.626	24		
C4	0.22	3.6472	26		
C5	0.89	14.754	30		

1 $$C=\frac{Q}{2\pi f{V}^{2}}$$

**Figure 2:**
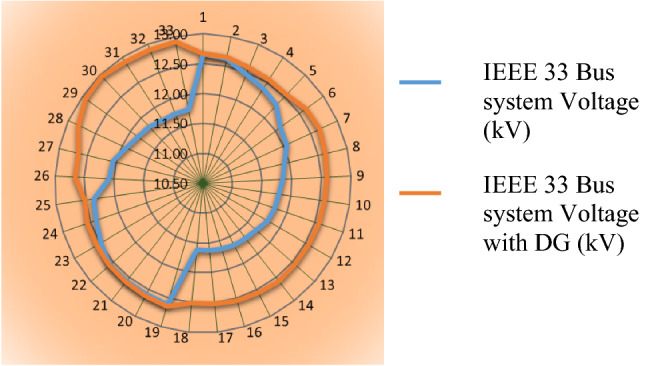
Voltage values of system busses before and after the integration of DGs.

Figure 2 shows the voltage on all buses before and after DG integration. It was clear from Fig. 2 that the role of DGs in supporting the voltage value of all system busses was near the rated value.

### Investigation of ferroresonance

The main causes of ferroresonance activation are the abnormal switching and faults. They may result in the interaction of nonlinear inductance with system capacitance. In this section, the actions that investigated ferroresonance in the modified IEEE system will be scrutinized.

### Load study

First, all loads linked to the capacitors were assessed, after which all abnormal separations on the distribution transformer terminals were investigated, resulting in ferroresonance investigations in two cases.

**Figure 3:**
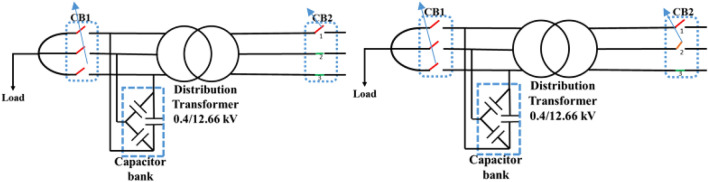
Separation and connection of load and transformer phases on HV side.

**Figure 4:**
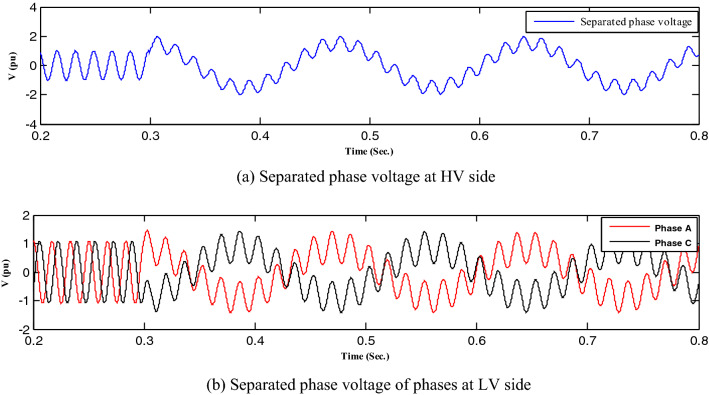
Ferroresonance at case one of separating CB1 and any phase of CB2.

**Figure 5:**
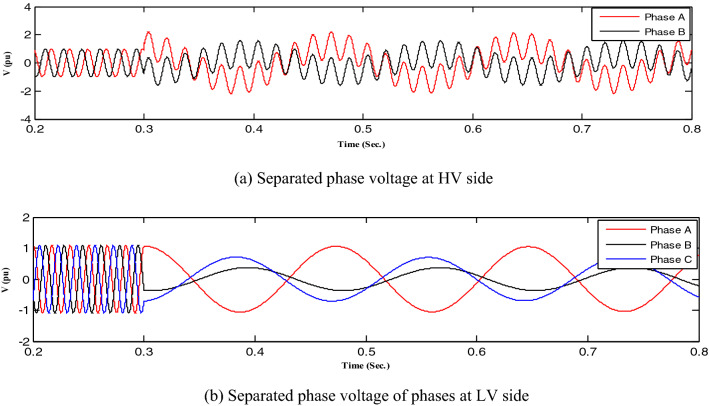
Ferroresonance at case two of separating CB1 and any two phases of CB2.

- Case one: the load was shaded, indicating a failure on a single-phase from the high-voltage side. Therefore, this case was modeled by separating CB1 and any phase of CB2, as shown in Fig. 3. While this model resulted in subperiodical ferroresonance in the separated phase on the high-voltage side of the transformer, it resulted in two phases on the low voltage side. Additionally, Fig. 4 shows the voltage wave preceding and following the separation moment. Although on the high-voltage side, the voltage value increased to 1.98 pu, on the low voltage side, it increased to 1.45 pu.
- Case two: the load was also shaded, failing in the two phases from the high-voltage side. Therefore, this case was modeled by separating CB1 and any two phases of CB2, as shown in Fig. 3. While this modeling resulted in subperiodical ferroresonance in one phase of the separated phases on the high-voltage side with a 2.19 pu value, the other separated phase on the high-voltage side of the subharmonic ferroresonance appeared with a 1.7 pu value. This subperiodical ferroresonance resulted in a low voltage of 1 pu value in all phases. Figure 5 depicts the voltage wave preceding and following the separation moment of the unhealthy phases.

### DG study

**Figure 6:**
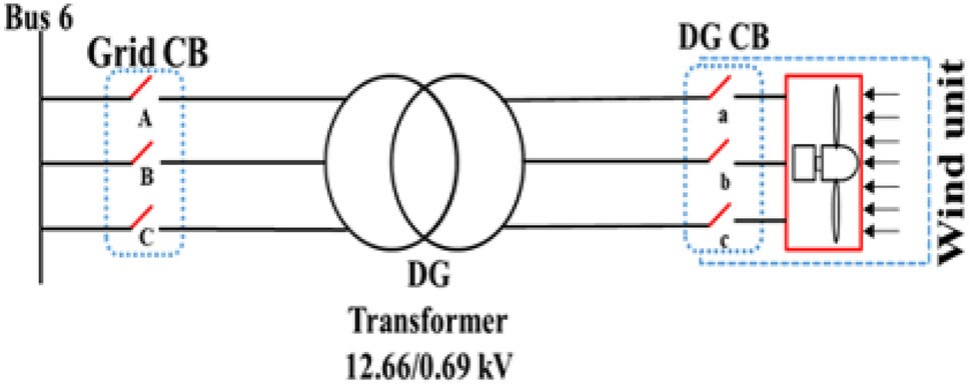
Schematic showing the connection of wind DG to the system.

**Figure 7:**
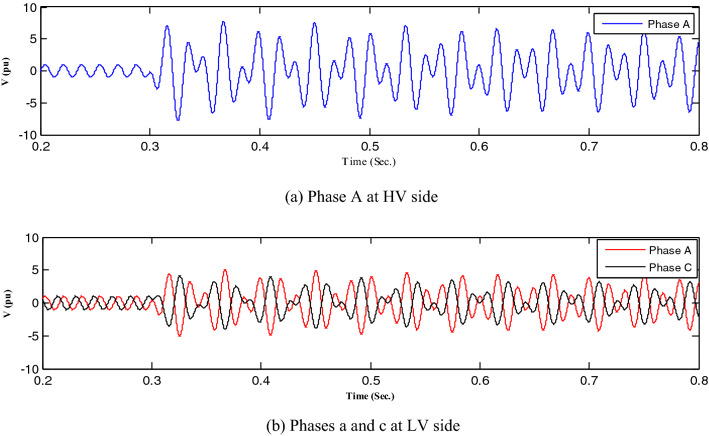
Ferroresonance at event 3.

**Figure 8:**
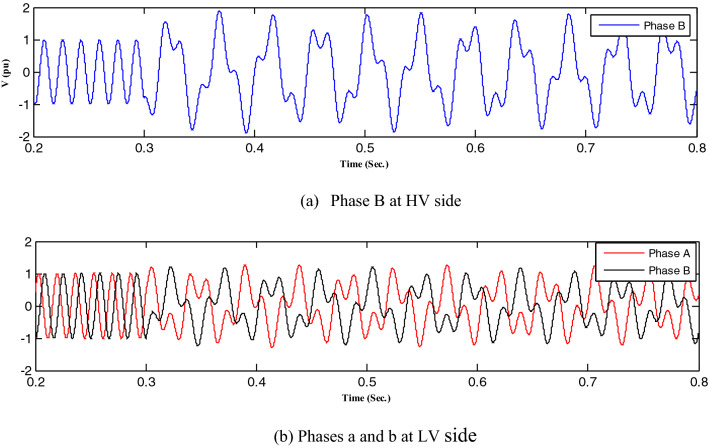
Ferroresonance at event 8.

**Figure 9:**
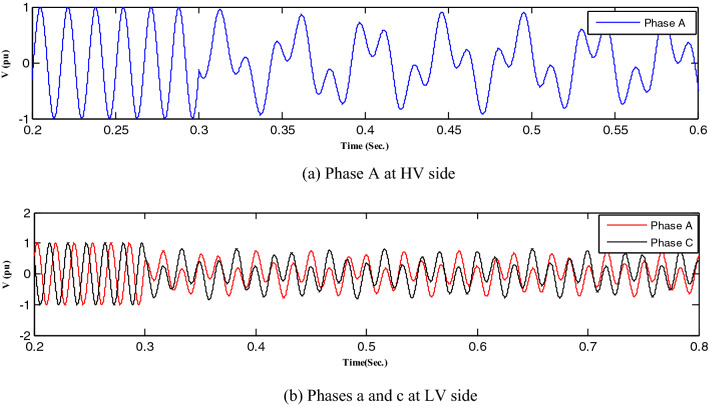
Ferroresonance at event 9.

**Figure 10:**
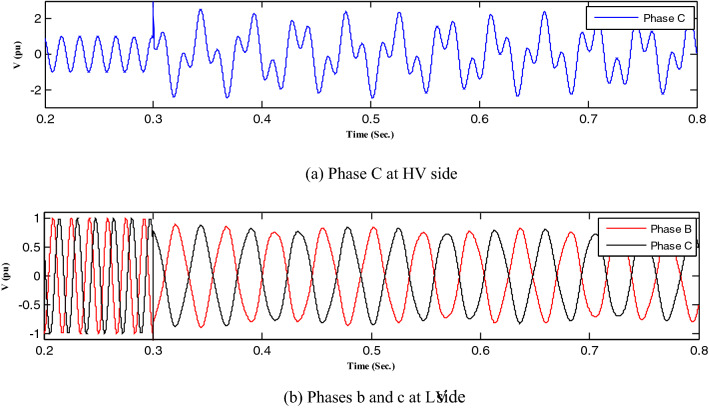
Ferroresonance at event 17.

**Figure 11:**
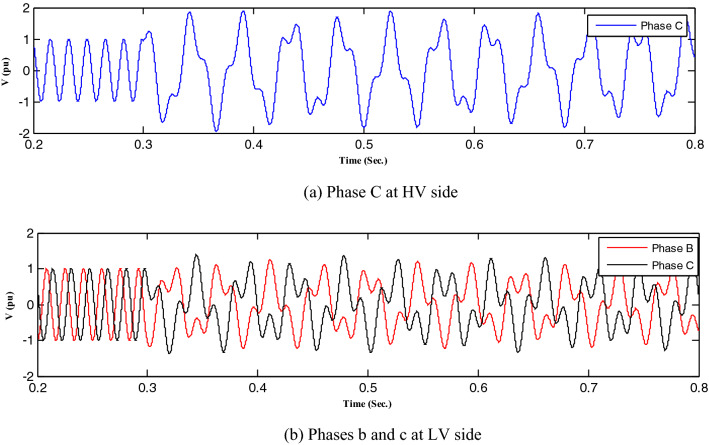
Ferroresonance at event 21.

**Figure 12:**
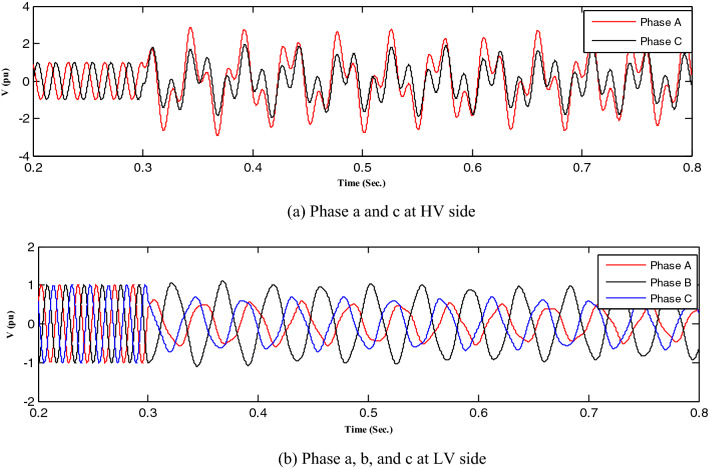
Ferroresonance at event 24.

All abnormal separation conditions on the wind DG integrated into the modified IEEE 33-bus system were studied in this work. Figure 6 indicates the connection of the wind DG to the understudied system. Investigations revealed that all series fault conditions resulted in ferroresonance, as summarized in Table 2. The events from 1 to 3 show a breakdown of similar phases on both sides of the transformer, which resulted in Quasi-Periodic Ferroresonance (QPF) on both sides of the DG transformer. The outcomes of these events were similar, and that of event 3 was presented as a representative example of this stage in Fig. 7. In contrast, the events from 4 to 9 showed the breakdown of unsimilar phases at both sides of the transformer. Although they also resulted in QPF on both sides of the DG transformer, and the outcomes of these events were similar, as shown in Fig. 8, the outcome of event nine differed, as shown in Fig. 9. The events from 10 to 18, however, indicate the breakdown of two phases on the high-voltage side and one phase on the low voltage side of the DG transformer. Notably, although they resulted in QPF on the high-voltage side of the DG transformer, they also resulted in a Sub Harmonic Ferroresonance (SHF) on the low voltage side of the DG transformer. In addition, the outcomes of these events were similar. Event 17 is presented as a representative example of this stage in Fig. 10. Remarkably, the events from 19 to 21 show that the breakdown of one phase on the high-voltage side of the DG transformer resulted in SHF on both sides of the DG transformer, also indicating that the outcomes of these events were similar. Event 21 is presented in Fig. 11. Finally, the events from 22 to 24 represent the breakdown of two phases on the high-voltage side of the DG transformer. Event 24 is presented in Fig. 12. Table 2 shows all separation arrangements, including their ferroresonance types and voltage values.

**Table 2:**
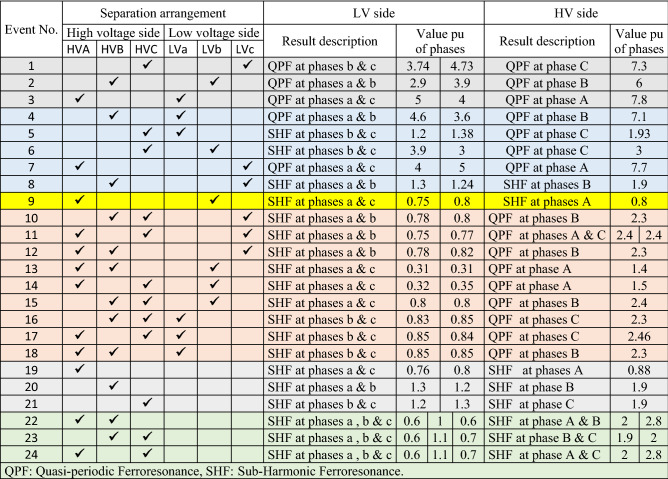
Summary of abnormal separation events of a IEEE 33-bus system penetrated by DG.

It can be observed that they resulted in SHF on both sides of the DG transformer, with the outcomes of these events being similar. It was evident that the resultant voltage value and shape did not change as the fault time varied.

### Mitigation of ferroresonance

Ferroresonance results in a substantial increase in current and/or voltage, which is posed a serious risk to the power network components. For this reason, the researchers concentrated on lowering the frequency of this phenomena in order to prevent its significant technical and financial issues. The RLC-SL as a ferroresonance mitigation technique is presented in this section, along with its design procedures.

### The proposed mitigation strategy

**Figure 13:**
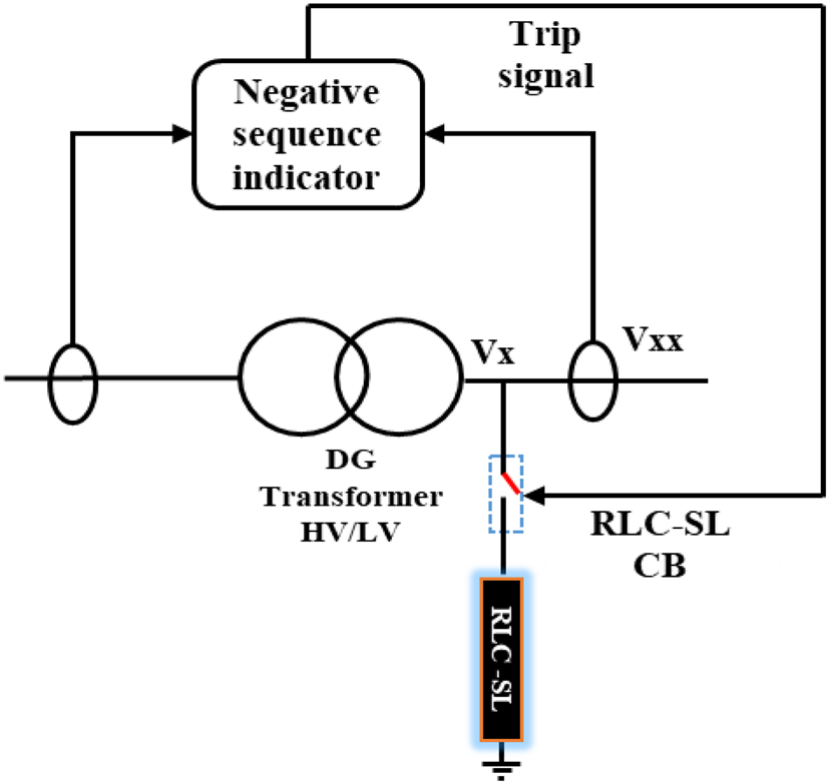
Position and mechanism of RLC-SL connection.

This section proposes an RLC-SL as a new method for mitigating the investigated ferroresonance. The proposed RLC-SL method was first compared with the shunt resistance method used in^32^, the shunt reactor method used in^33^, the series resistance method used in^34^ and the shunt nonlinear reactor method used in^35^. Notably, the proposed RLC-SL method is a shunt RLC that connects to the transformer's low voltage side. As Fig. 13 indicates, the RLC-SL was linked by a breaker that receives a trip signal from the negative sequence detector. Moreover, during the ferroresonance state, the negative sequence had a value greater than zero. Therefore, changes in the negative sequence value could be used to activate the breaker, as Fig. 13 also demonstrates. To this end, the proposed RLC-SL was used to mitigate the ferroresonanc wave to the closest form of the steady-state voltage. Equation 2 demonstrates the mathematical model used to obtain a steady-state voltage.

2 $${V}_{sd}=\left|{V}_{max}\right|\mathrm{sin}(2\pi ft)$$

where V_sd_ is the steady-state voltage, V_max_ is the maximum voltage, and f is the system frequency.

**Figure 14:**
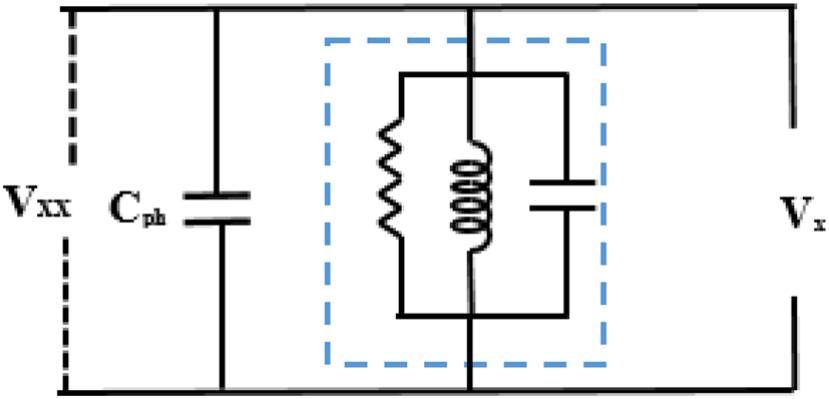
Equivalent circuit at the load stud applying the RLC-SL circuit.

Subsequently, the effect of the RLC-SL could be deduced from the equivalent circuit in each case. The first case was the load study, with the equivalent circuit after the separation event being shown in Fig. 14. As a result, the equivalent circuit could be used to derive the Eqs. (3)–(13) that express steady-state voltage values by applying the proposed RLC-SL method to show how the changes in RLC parameter values effect on the value of ${Z}_{RLC}$, subsequently changing the voltage wave.

3 $${V}_{xx}={I}_{Cph}\times {x}_{Cph}$$

where *V*_*xx*_ is the voltage wave after inserting RLC-SL, *I*_*cph*_ is the capacitor bank's single-phase current, and X_cph_ is the capacitor bank’s single-phase impedance.

4 $${I}_{Cph}={I}_{Source}-{I}_{RLC}$$

where *I*_*source*_ is the current from the source, and *I*_*RLC*_ is the current through the RLC-SL.

5 $${I}_{Source}=\frac{{V}_{x}}{{Z}_{Total}}$$

where* V*_*x*_ is the voltage wave before adding RLC-SL, and *Z*_*Total*_ is the equivalent impedance of RLC-SL and the capacitor bank’s single-phase impedance.

6 $${I}_{RLC}=\frac{{V}_{x}}{{Z}_{RLC}}$$

where* I*_*RLC*_ is the RLC-SL current, and *Z*_*RLC*_ is the total impedance of RLC-SL

7 $${V}_{xx}={V}_{x}\times {x}_{Cph}\times \frac{{Z}_{RLC}-{Z}_{Total}}{{Z}_{RLC}\times {Z}_{Total}}$$

8 $${C}_{ph }={\left[\frac{{{C}_{delta}}^{-2}}{3{{C}_{delta}}^{-1}}\right]}^{-1}$$

where* C*_*ph*_ is the capacitor bank phase value, and *C*_*delta*_ is the capacitor bank-line value.

9 $${x}_{Cph}=\frac{1}{2\pi f{c}_{ph}}$$

10 $${Z}_{RLC}=\frac{R\times {X}_{C}\times {X}_{L}}{\left(R\times {X}_{C}\right)+\left(R\times {X}_{L}\right)+\left({X}_{L}\times {X}_{C}\right)}$$

where* R* is the resistance value of RLC-SL, *X*_*c*_ is the capacitor reactance value of RLC-SL, and X_L_ is the reactance value of RLC-SL.

11 $${x}_{C}=\frac{1}{2\pi fC}$$

where* C* is the capacitor value of RLC-SL.

12 $${X}_{L}=2\pi fL$$

where L is the inductance value of RLC-SL.

13 $${Z}_{RLC}=\frac{i\omega L}{1-LC{\omega }^{2}+\frac{i\omega L}{R}}$$

14 $$\omega =2\pi f$$

**Figure 15:**
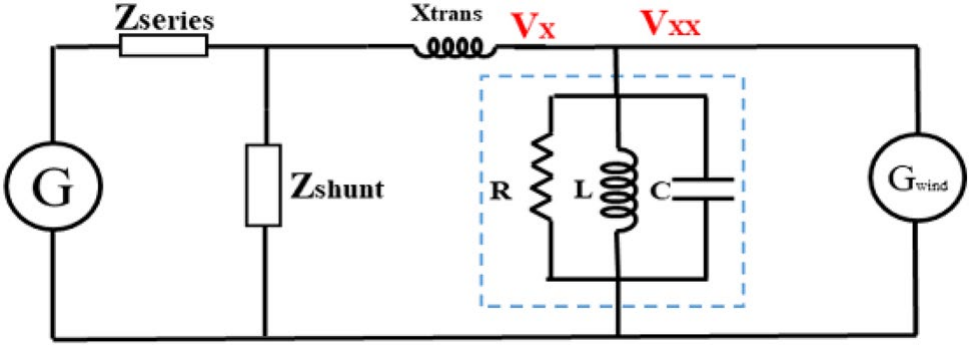
The equivalent circuit at DG study after adding RLC-SL.

The second case included a wind generator as the DG, with the equivalent circuit after a series fault indicated in Fig. 15. Notably, the equivalent circuit could be used to derive Eqs. (8)–(16) that express the value of ${V}_{xx}$ after the addition of RLC-SL, where *I*_*Load1*_ is the load current from the DG, and ${I}_{Wind}$ is the DG current. Then, since the load was constant, changes in the RLC values changed the *Z*_*RLC*_ and *I*_*RLC*_ values, which resulted in *V*_*xx*_ value changes. Hence, by tracking the change in V_xx_, the closest state to *V*_*sd*_ could be determined.

15 $${V}_{xx}={I}_{RLC}\times {Z}_{RLC}$$

16 $${I}_{RLC}={I}_{Wind}-{I}_{load1}$$

### Design of the RLC-SL

**Figure 16:**
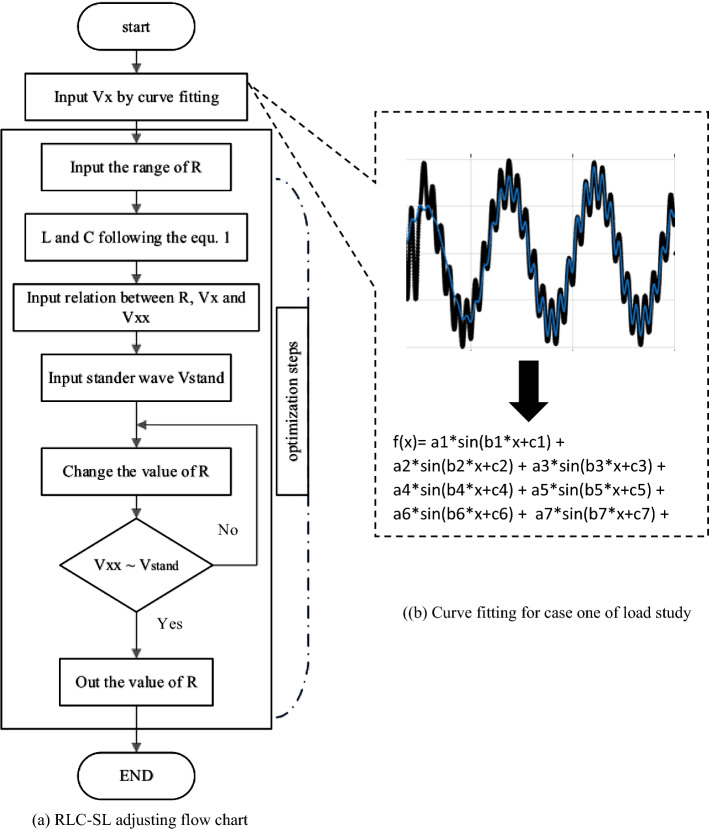
RLC-SL adjusting steps.

Adjusting the RLC-SL value is divided into two steps: adjusting the LC together and optimizing the RLC values are tuned according to Eq. (17). The procedures for obtaining the desired value for *R*, are shown in Fig. 16. The design process begins with entering the voltage wave equation in the ferroresonance condition by curve fitting, then entering the acceptable range of *R.* The values are modified and updated until reaching the optimal values using a designed procedure which indicated by the flow chart of Fig. 16. The value of *R* is inferred by compensating with the many accepted values of *R* and comparing the shape of the resulting wave in each value with the voltage wave in the steady-state.

The value of *R* is chosen that drives the voltage waveform as closely as possible to the steady-state wave. This study is carried out using MATLAB software. The ferroresonance voltage wave was initiated by a MATLAB m-file implementing the curve fitting tool. The values of the RLC are determined by employing the previously described Eqs. (2)–(18). Figure 16a demonstrates the flow chart of the design procedure implemented in selecting the parameters of the proposed mitigation scheme. Figure 16b illustrates a curve fitting example for the load study in case one. As a result, the proper RLC-SL value for any system can be determined. The effectiveness and efficacy of the proposed scheme will be demonstrated by comparing its result with those obtained using other existing ferroresonance mitigation methods used in the literature.

17 $$\omega =\frac{1}{\sqrt{LC}}$$

where* L* and *C* are the capacitor and inductance values of RLC-SL.

### Comparison of mitigation strategies

**Figure 17:**
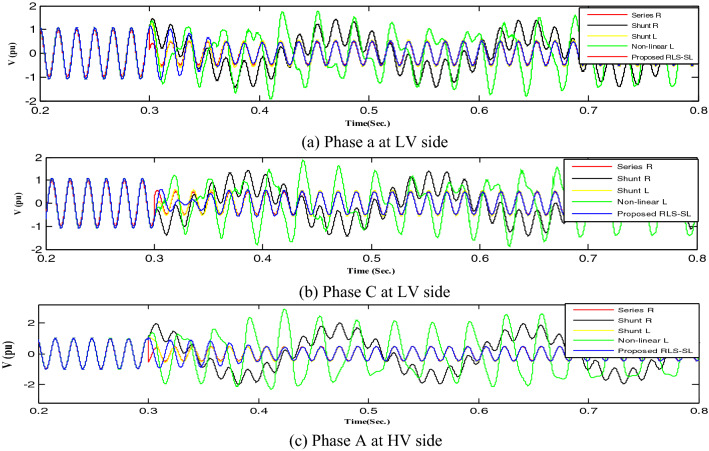
Mitigation ferroresonance at case one of load study.

**Figure 18:**
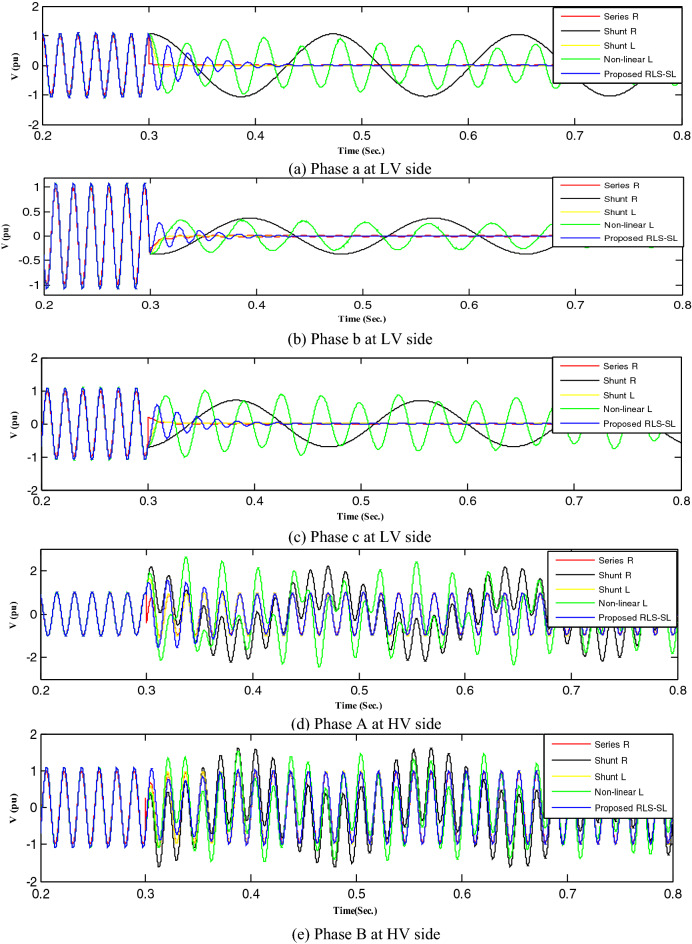
Mitigation ferroresonance at case two of load study.

**Figure 19:**
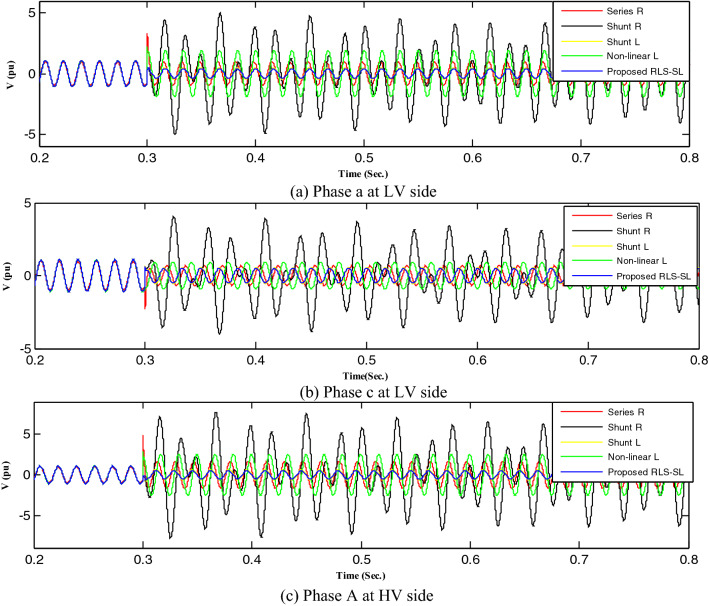
Mitigation ferroresonance at event 3.

**Figure 20:**
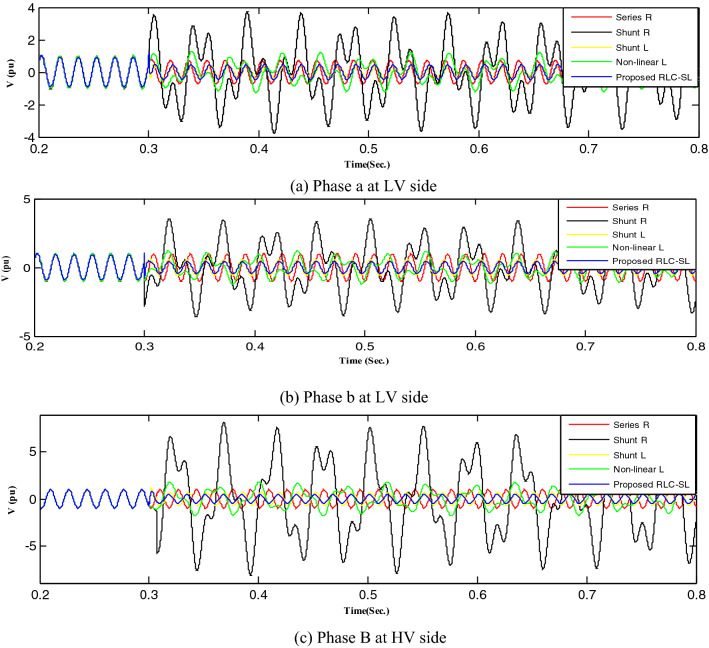
Mitigation ferroresonance at event 8.

**Figure 21:**
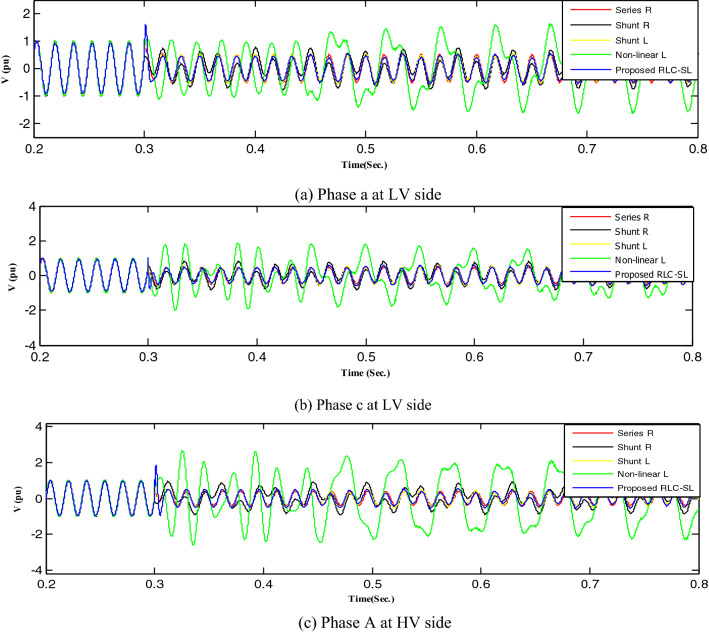
Mitigation ferroresonance at event 9.

**Figure 22:**
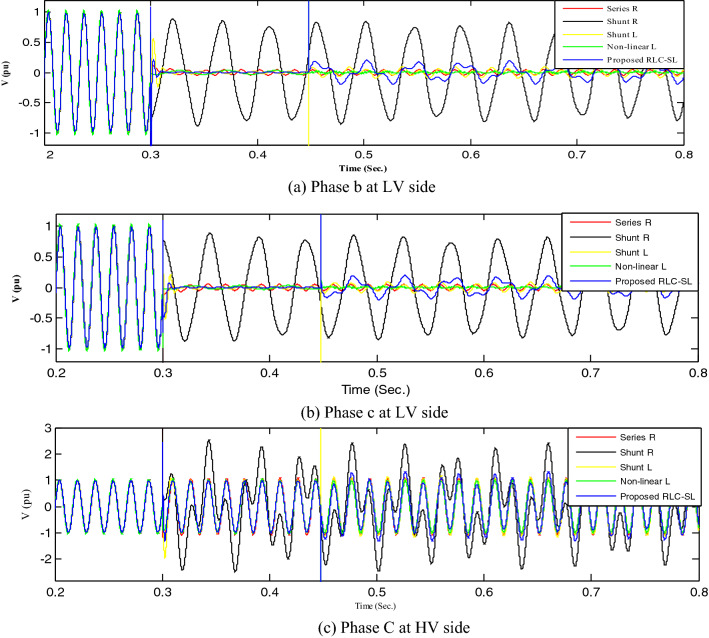
Mitigation ferroresonance at event 17.

**Figure 23:**
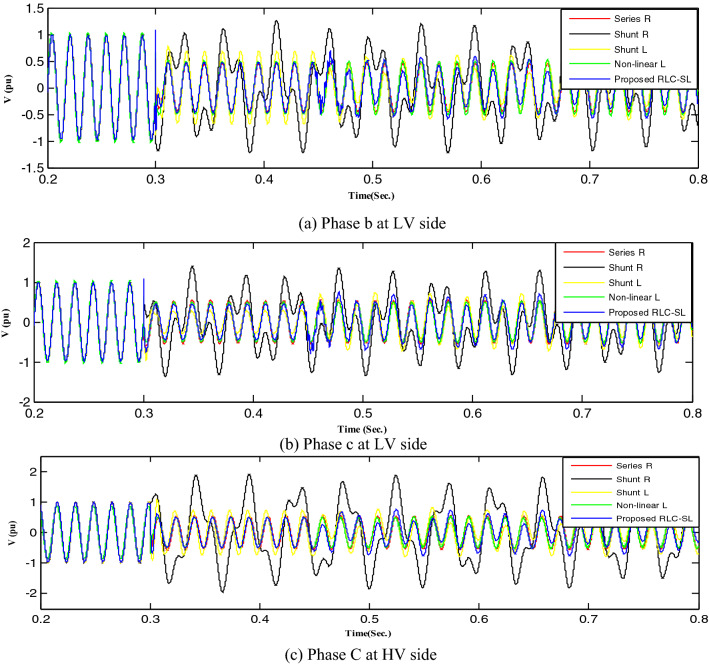
Mitigation ferroresonance at event 21.

**Figure 24:**
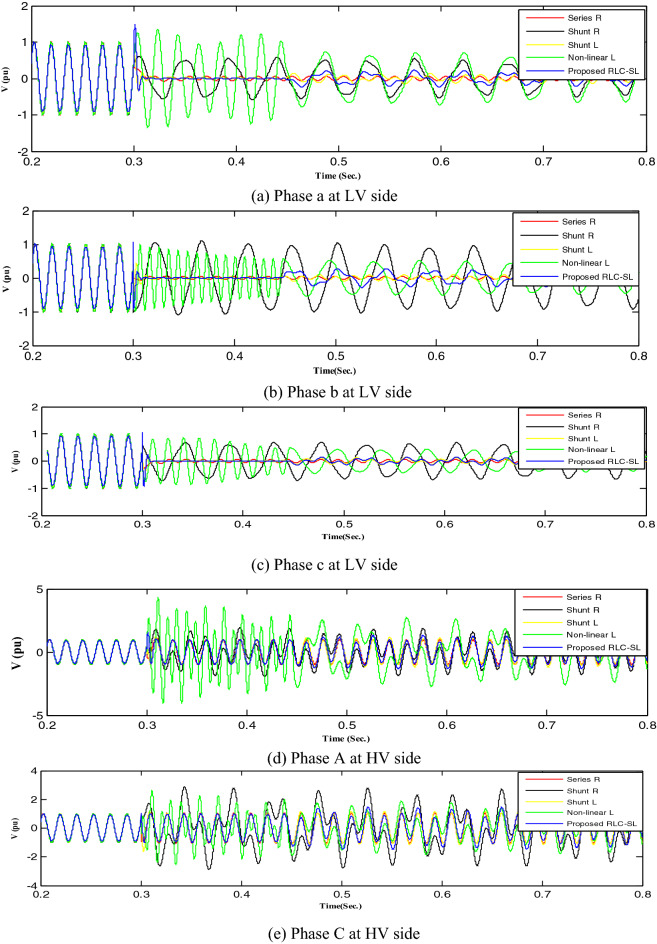
Mitigation ferroresonance at event 24.

This section implemented and compared the previously published mitigation methods with the proposed method. Therefore, the shunt resistance^36^, shunt reactor, nonlinear shunt reactor, series resistance, and RLC-SL were implemented, with each technique result indicated by a distinct color (shunt resistance was represented by black, shunt reactor was represented by yellow, series resistance was represented by red, nonlinear inductor was represented by green, and the proposed RLC-SL was represented by blue). While the results using ferroresonance mitigation techniques for the first and second load study cases are shown in Figs. 17 and 18, the results of implementing mitigation techniques with the wind unit at ferroresonance conditions are indicated in Figs. 19, 20, 21, 22, 23 and 24.

By implementing the shunt resistance method, cases 1 and 2 of the load showed no effective mitigation, as shown in Figs. 17 and 18, whereas in the wind unit study, effective mitigation was observed, as Figs. 19, 20, 21, 22, 23 and 24 indicate. As a result, it was regarded as a pointless solution.

Contrastively, by implementing the shunt nonlinear reactor method, although effective mitigation in cases 1 and 2 of the load was observed, it was not a good solution because of the high distortion in the voltage form, as shown in Figs. 17 and 18. Moreover, in the wind unit study, while no effective mitigation was observed, as Figs. 19, 20, 21, 22, 23 and 24 indicate; some phases were distorted, such as events 12 and 13, as depicted in Figs. 19, 20, 21, 22, 23 and 24.

Additionally, effective mitigation was observed in the wind unit study by implementing the shunt reactor method, as shown in Figs. 19, 20, 21, 22, 23 and 24, whereas in the load study, there was no effective mitigation, as Figs. 17 and 18 indicate.

However, by implementing the series resistance method, although cases 1 and 2 showed effective mitigation, as shown in Figs. 17, 18, 19, 20, 21, 22, 23 and 24, it was regarded as an uneconomical solution because it resulted in permanent system losses.

Hence, looking at this method's economic feasibility, the transformer's average lifetime should range from 25 to 40 years^37,38^. Supposing the average lifetime is 35 years, this period is equivalent to 302,400 h. Besides, according to the Global Energy Institute, while the cost of a kilowatt-hour in the United States is 11.18 cents, or 0.1181$, the cost of losses due to the passage of only 1A from a wind unit was 1071.5 $, which is 557 $ at the load. As a result, the total cost of losses exceeds the cost of the transformers required to protect them from ferroresonance. Therefore, it is preferable to avoid using this technique. Remarkably, implementing the proposed RLC-SL method showed that cases 1 and 2 were effective in mitigation, as shown in Figs. 17, 18, 19, 20, 21, 22, 23 and 24. While the green cells indicate that the voltage in that phase was normal and not affected by series faults, the red cells indicate that the voltage at that phase was distorted after applying the technique. Table 3 also depicts the efficacy of the proposed RLC-SL in comparison with the series resistance, and shunt reactor in mitigating ferroresonance. As previously stated, the shunt reactor failed to mitigate ferroresonance during load studies, proposing series resistance as an uneconomical solution. As a result, the proposed RLC-SL was the most effective solution for ferroresonance mitigation. Table 3 presents the maximum output voltage values for each phase after all techniques had been applied to the wind.

**Table 3:**
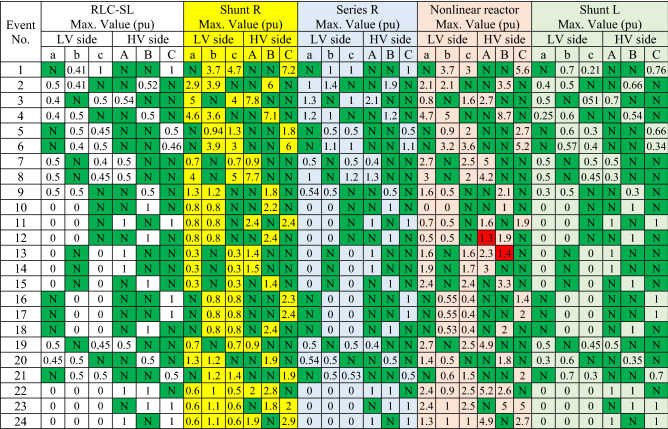
Summary of mitigation techniques results.

## Conclusion

Ferroresonance is a hazardous condition caused by the series association of equivalent capacitance and nonlinear inductance. It can result in sustained overvoltage damaging equipment. Therefore, this paper discussed the research gap point, represented by ferroresonance verification studies in DS penetrated by DGs. Then, scenarios that could lead to the IEEE 33-bus DS integrated with multi-DGs to ferroresonance were presented, after which the conditions of ferroresonance and their consequences were confirmed by simulating the DS in PSCAD/EMTDC. Finally, a method was proposed for mitigating ferroresonance in distribution networks using RLC-SL, after which a technique for determining the RLC values was provided that fit the modified IEEE-33 bus DS and can be applied to any other system. During ferroresonance, the proposed RLC-SL was connected to the system via a controllable switch that takes a trip signal from the negative sequence detector. Hence, the designed RLC-SL was evaluated in comparison to the shunt resistor, shunt nonlinear reactor, shunt linear reactor, and series resistance. Remarkably, the proposed method outperformed the other methods in terms of ferroresonance mitigation efficiency.

## Data Availability

The data that support the findings of this study are available from the corresponding author upon reasonable request.
